# In vitro wound healing effects of postbiotics derived from the gut microbiota of long‐lived blind mole rats, a model of healthy ageing

**DOI:** 10.1111/wrr.70023

**Published:** 2025-04-18

**Authors:** Hazal K. Demirhan, Zeynep B. Aksoy, Basar Karaca, Teoman Kankilic, Kamil C. Akcali, Fadime Kiran

**Affiliations:** ^1^ Pharmabiotic Technologies Research Laboratory, Department of Biology, Faculty of Science Ankara University Ankara Turkey; ^2^ Graduate School of Natural and Applied Sciences Ankara University Ankara Turkey; ^3^ Stem Cell Institute Ankara University Ankara Turkey; ^4^ Microbiology Research Laboratory, Department of Biology, Faculty of Science Ankara University Ankara Turkey; ^5^ Department of Biotechnology, Faculty of Science Nigde Omer Halisdemir University Nigde Turkey; ^6^ Department of Biophysics, Faculty of Medicine Ankara University Ankara Turkey

**Keywords:** biofilms, infection, inflammation *Limosilactobacillus reuteri*, postbiotics, proliferation, wound healing

## Abstract

Chronic wounds represent a global public health burden to patients and healthcare professionals worldwide. Considering the unmet need for safe and effective therapeutic approaches for wound healing, research on discovering new bioactive materials that support all stages of wound healing is gaining importance. In this study, the wound‐healing activity of postbiotics obtained from *Limosilactobacillus reuteri* EIR/Spx‐2, isolated from the gut microbiota of long‐lived blind mole rats (*Nannospalax xanthodon*), was investigated. Our results demonstrated that postbiotics exhibited a strong inhibitory effect against important skin pathogens, eliminated their biofilm formation, and downregulated the expression of genes involved in their quorum‐sensing regulatory mechanisms. Furthermore, treatment with postbiotics resulted in a significant increase (23.82% ± 2.11%) in L929 fibroblast cell proliferation. Additionally, postbiotics applied on scratched fibroblast monolayer significantly accelerated the re‐epithelialization by 66.78% ± 3.74%. The treatment also increased the mRNA expression and protein levels of COL1A1 in the early healing phase. Moreover, the intracellular ROS levels of L929 cells suppressed by H_2_O_2_ were significantly reduced, which could be attributed to the content of flavonoids (4.8 mg/g) and phenolic compounds (7.12 mg/g) in postbiotics, as well as their DPPH scavenging activity. After treatment with postbiotics, the mRNA levels of IL‐6 (5.77‐fold) and TNF‐*α* (1.76‐fold) and the amount of NO (79.25% ± 3.18%) were significantly decreased in LPS‐induced murine macrophages. The diverse metabolite profile of postbiotics, as characterised using chromatographic techniques, exhibited a strong correlation with their biological activity across all stages of the wound healing process, highlighting their potential as promising candidates for wound healing applications.

**Abbreviations**
agrA: accessory gene regulator A
cDNA: complementary deoxyribonucleic acid
COL1A1: collagen type I alpha 1 chain
DPPH: 2,2‐diphenyl‐1‐picrylhydrazyl
GAPDH: glyceraldehyde 3‐phosphate dehydrogenase
H_2_O_2_
: hydrogen peroxide
hld: delta‐lysin gene
HRP: horseradish peroxidase
IL: interleukin
LPS: lipopolysaccharide
mRNA: messenger ribonucleic acid
NaOH: sodium hydroxide
NO: nitric oxide
PCR: polymerase chain reaction
PVDF: polyvinylidene difluoride
ROS: reactive oxygen species
THP: Tohoku Hospital Paediatrics‐1
TNF‐*α*
: tumour necrosis factor‐alpha

## 1 INTRODUCTION

In recent years, the incidence of wounds, particularly chronic wounds, has increased rapidly as a silent epidemic, which leads to a global public health burden on patients and healthcare systems. According to current reports, 1%–2% of people in industrialised countries will experience chronic wounds at least once in their lifetime.^1^ In the USA alone, over 10.5 million patients suffer from chronic wounds, costing the healthcare system US$28 billion annually.^2^ Moreover, it has also been reported that 1.5–2 million people suffer from acute or chronic wounds and occupy 25%–50% of hospital beds in Europe.^3^ The alarming increase in the prevalence of chronic wounds is related not only to the rapidly growing geriatric population but also to the significantly increasing prevalence of comorbidities and lifestyle diseases such as diabetes, obesity, and venous hypertension.^3^ By 2050, there will be more than 1.31 billion people with diabetes,^4^ and the elderly population will reach more than 2.1 billion,^5^ suggesting that chronic wounds will continue to be an increasingly persistent problem worldwide. With this regard, chronic wounds are expected to remain a significant clinical, social, and economic issue in the future.^6^ To reduce morbidity and mortality, as well as alleviate the financial burden on patients, innovative wound care products that offer improved healing outcomes and promote a faster recovery process are still gaining increased attention.

A comprehensive understanding of the wound healing process, which consists of four overlapping phases involving haemostasis, inflammation, proliferation, and remodelling, is crucial to pave the way for accelerated wound healing.^7^ Current strategies such as physical closure of the wound margin, sutures, and dressings are mainly used to heal chronic wounds and achieve skin tissue regeneration. Over the past few decades, natural bioactive components with anti‐inflammatory, antioxidant, antibacterial, and collagen‐promoting effects have been integrated into these therapies as wound‐healing agents.^8^ However, there are still significant challenges in using natural compounds derived from plant or animal sources, such as containing toxic solvent phases in their production process, being thermolabile, and leading to irritation or allergic reactions.^9^, ^10^ Therefore, the wound care industry, estimated to reach $22 billion by 2030,^11^ has recently focused on novel and natural wound care compounds that are particularly effective in bacterial burden management and biological therapies. Nonetheless, the need for effective wound treatment solutions that accelerate each phase of the wound healing process and address the limitations of the industry remains a significant issue.

To date, numerous in vitro and in vivo experiments and clinical trials have highlighted the beneficial effects of probiotic formulations on wound repair.^12^ Notably, wound dressings^13^ or hydrogels^14^ incorporating probiotics possess appropriate properties for wound management by protecting against pathogenic agents, modulating inflammatory responses, regulating collagen deposition, and stimulating angiogenesis. Despite their strong impact on the wound‐healing process, probiotics may not remain viable during the production, storage, or delivery of the final product.^15^ Therefore, the application of probiotics in wound healing products faces significant commercial and technological challenges related to the maintenance of their cellular viability. Moreover, safety concerns regarding the overuse of living cells in immunocompromised patients and infants have also attracted attention.^16^ To overcome these disadvantages, the application of metabolites or fragments derived from probiotics (postbiotics) has garnered recent interest as a novel therapeutic and preventive strategy in modern medicine. In mid‐2021, the International Scientific Association for Probiotics and Prebiotics (ISAPP) defined the postbiotic as, ‘a preparation of inanimate microorganisms and/or their components that confers a health benefit on the host’^17^ Based on this definition, postbiotics can contain microbial cell fractions, cell lysates, short‐chain fatty acids (SCFAs), extracellular polysaccharides (EPS), teichoic acid, organic acid, proteins, enzymes, and muropeptides derived from peptidoglycan. Since postbiotics do not contain live cells, their usage lacks any possible side effects of probiotics. Importantly, they maintain beneficial effects on wound healing through similar mechanisms to those of probiotics. These mechanisms include regulation of the immune response, modulation of the skin microbiota, inhibition of pathogens, and strengthening of the epithelial barrier.^18^ Therefore, postbiotics have recently attracted interest in wound management strategies with their broad‐scale modulatory effects. Nevertheless, the selection of the ideal postbiotic‐producer strains requires special attention depending on the target case because of that their efficacy largely depends on the bacterial strain. Consequently, recent studies have focused on discovering novel strains with multi‐functional properties and their incorporation into next‐generation products.

In this regard, the present study aimed to investigate the potential effects of postbiotics derived from *Limosilactobacillus reuteri* EIR/Spx‐2 (*L. reuteri*) on wound healing under in vitro conditions. This strain was isolated from the gut microbiota of long‐lived blind mole rats, *Nannospalax xanthodon* (*Spalax*). The extraordinarily long life spans of >20 years and natural resistance to age‐related pathologies make these animals attractive for ageing research.^19^ Based on this information, we hypothesised that their gut microbiota might be potentially linked to their extraordinary longevity and healthy ageing; thus, their microbiota‐derived metabolites could be used as a possible age modulator for further biomedical research.^20^, ^21^ To the best of our knowledge, this is the first study on the transition of the gut microbiota of *Spalax* to wound management strategies.

## 2 MATERIALS AND METHODS

### 2.1 Bacterial strains, cells, and their culture conditions

*Pseudomonas aeruginosa* PAO1/ATCC 27853, methicillin‐resistant *Staphylococcus aureus* (MRSA) ATCC 43300, and *Staphylococcus epidermidis* ATCC 35894 were purchased from the American Type Culture Collection (ATCC) and used for antimicrobial, anti‐biofilm, and anti‐quorum sensing (QS) experiments. Unless otherwise stated, the important skin pathogens were grown in TSB (Tryptic Soy Broth, Merck, Germany) media for 24 h at 37°C. All bacterial strains were maintained in 50% glycerol (v/v) at −80°C.

NCTC clone 929 [L cell, L‐929, derivative of Strain L, murine fibroblast] was kindly purchased from the Veterinary Faculty of Ankara University (Ankara, Turkey) and cultured in high glucose Dulbecco's modified Eagle medium (DMEM, Gibco, USA) supplemented with 10% fetal bovine serum (FBS, Sartorius, Germany), 1% antibiotic‐antimycotic solution (Sartorius, Germany), and 1% nonessential amino acids (Gibco, USA) in 75 cm^2^ culture flasks (Sarstedt, Germany) under a 5% CO2 and 95% humidified atmosphere at 37°C. The murine monocyte/macrophage‐like cell line RAW 264.7 (ATCC TIB‐71), a kind gift from Prof. Dr. Ihsan Gursel (THORLab, IBG, Izmir, Turkey) was used for immunological assays. The RAW 264.7 cells were cultured under similar media and conditions as described previously. After the cells reached 80% confluence in the flasks, they were rinsed with 1× phosphate‐buffered saline (PBS, Sartorius, Germany), harvested with 0.25% trypsin–EDTA (Sartorius, Germany), and centrifuged at 200 × *g* for 5 min. The cells were then stained with trypan blue (Sartorius, Germany), counted with a TC20 Automated Cell Counter (Bio‐Rad, USA), and used for further analysis. All the experiments were performed using the same FBS batch, and the cells were regularly tested for bacteria, fungi, and mycoplasma contamination using a commercial PCR‐based kit (ThermoScientific, USA) during the experimental protocols.

### 2.2 Animals

*N. xanthodon* (*n* = 3 female, *n* = 3 male) blind mole rats (2*n* = 58) were caught with a metal pipe‐type trap from the countryside of Nigde (Central Anatolia region, Turkey). Morphometric observations were performed for their identification, and chromosome numbers were confirmed by karyotyping according to Ford and Hamerton (see Supplementary Figure 1).^22^ Animals were immediately brought to the laboratory under aseptic conditions, cleaned by copiously spraying with 80% ethanol, and then dissected after euthanasia by CO_2_ inhalation. Fresh faecal samples collected from the colonic contents were used as the starting materials for bacterial isolation.

The protocols employed were approved by the Nigde Omer Halisdemir University Local Ethics Committee on Animal Experimentation (Approval no: 3190652/050.99) with the permission of the Republic of Turkey, Ministry of Forest and Water Works.

### 2.3 Isolation and identification of culturable commensal bacteria

Commensal bacterial strains were isolated from fresh faecal samples of healthy blind mole rats. Each sample was homogenised in 1.0 mL of phosphate‐buffered saline (PBS; pH 7.2), and a tenfold dilution series of the suspensions was spread plated on Man Rogosa Sharpe (MRS, Merck, Germany) agar. After an incubation period of 48–72 h under aerobic conditions at 37°C, three colonies with distinct morphologies from those of *Lactobacillus* spp. genera were selected from each stool sample. Pure isolates with Gram‐positive and catalase‐negative phenotypes were selected and then stored in a sterile glycerol/MRS broth mixture (50:50%; v/v) at −80°C for further analysis.

Molecular identification of the isolates was determined by sequencing the 16S ribosomal‐RNA (*16S rRNA*) subunit gene region of the bacterial genome. In brief, genomic DNA was isolated using a commercial kit (Qiagen, Germany) according to the manufacturer's instructions, and the *16S rRNA* gene was amplified by polymerase chain reaction (PCR, Thermal Cycler, Bio‐Rad, USA) using the universal primers (see Supplementary Table 1). The PCR product was purified from the agarose gel using a purification kit (Qiagen, Germany) and sequenced by the commercial services of AgriGenomics Hub (Ankara, Turkey). The sequences were then analysed using the Basic Local Alignment Search Tool (BLASTn) to identify the sequences with the highest nucleotide similarity within the GenBank database.

### 2.4 Preparation of microbiota‐derived postbiotics

The isolated strains were grown in 100 mL of MRS broth at 37°C for 24 h. After incubation, the culture supernatant was collected by centrifugation at 15.000 × *g* for 20 min, and sterile filtration was performed using a microporous membrane filter with a pore size of 0.22 μm (Sartorius, Germany) to completely remove the cells. The filtered supernatant was then lyophilized (freezing conditions of −20°C, a vacuum pressure of 0.120 mB and a condenser temperature of −58°C) using a freeze dryer (Buchi, Switzerland). The lyophilized powder was solubilised in sterile distilled water at a final concentration of 100 mg/mL, sterilised using membrane filters as described before and stored at −20°C until use.^23^

### 2.5 Screening of the isolates for their antimicrobial activity

The postbiotics were tested against important skin pathogens using the agar well diffusion assay.^23^ Briefly, pathogenic bacteria grown in TSB at 37°C for 24 h were adjusted to the 0.5 McFarland standard (Biomeireux, France), inoculated in soft agar, and then poured onto the agar surfaces. Postbiotics at a final concentration of 100 mg/mL were then transferred into the 6 mm apertures on agar surfaces. MRS medium was used as a negative control. Following the incubation period at 37°C for 24 h, the diameter of the inhibition zones around the wells was measured in mm.

To find out whether antimicrobial peptides or acid‐dependent end products played a role in the antibacterial activity against the pathogens, the inhibitory effect was also analysed following the treatment of the postbiotics with 1 mg/mL proteinase K and 1 mg/mL catalase. In addition, the retention of activity in the postbiotics following the exposure to heat (100°C for 15 min) and neutralisation with 1 N NaOH was also evaluated using the agar well diffusion method.^23^

The minimum inhibitory concentration (MIC) of the postbiotics against all pathogenic strains was determined using a microtiter plate assay according to Clinical and Laboratory Standards Institute guidelines.^24^ Briefly, twofold dilutions of postbiotics (1.024–0.25 mg/L) and an overnight culture of each pathogen adjusted to 0.5 McFarland were co‐incubated in each well of the microtiter plates. The cultures without postbiotics were used as a positive control, while the medium without pathogens was used as a negative control. Following the incubation period at 37°C for 24 h, the optical density in each well was measured at a wavelength of 620 nm using a microplate reader (BioTech, USA). The lowest concentration that completely inhibited bacterial growth was determined as the MIC value.

### 2.6 Assessment of anti‐biofilm activity

Biofilm formation was assessed in 96‐well polystyrene microplates (LP Italiana, Italy) using a crystal violet binding assay according to Stepanovic et al.^25^ with slight modifications. To induce biofilm formation, pathogens were cultured in modified media (TSB enriched with 0.5% glucose for *P. aeruginosa* PAO1/ATCC 27853, 3.0% NaCl and 3.0% glucose for MRSA ATCC 43300, and 1:10 diluted TSB for *S. epidermidis* ATCC 35894) as determined in our previous studies.^26^ Briefly, the cultures adjusted to the 0.5 McFarland standard were cocultured with different concentrations of postbiotics at 37°C for 24 h. After incubation, planktonic cells were removed and the wells were immediately fixed with 200 μL of 95% methanol for 15 min and stained with 200 μL of 0.1% crystal violet solution. After 30 min of incubation at room temperature, the wells were washed with distilled water to remove the unbound dye and then air‐dried. Finally, dye bound to the biofilm was dissolved with an acetone: ethanol (30:70 v/v) solution, and each biomass was quantified by measuring the absorbance at a wavelength of 620 nm using a microplate reader. Modified TSB media without pathogens were used as a negative control. Biofilm reduction was then analysed with the formula [(*C* − *B*) − (*T* − *B*)]/[(*C* − *B*)] × 100 (*C*, absorbance value of the well including the pathogen; *B*, absorbance value of the well including the modified medium; *T*, absorbance value of the well including the pathogen and postbiotics, together).

To evaluate the effects of postbiotics on the bacterial biofilm matrix microscopically, a biofilm assay was also performed on glass‐made coverslips, and the biofilms were analysed using a LIVE/DEAD BacLight Bacterial Viability Kit (L7007, Thermo Fisher, USA) according to the user manual. Imaging was performed with confocal laser scanning microscopy (CLSM, a Plan‐Neofluar 40X/1.3 DIC objective; Carl Zeiss Microscopy, Germany). The green and red fluorescence of SYTO 9 and propidium iodide (PI) was excited with an argon laser beam at 488 nm (5% intensity) and a helium/neon source at 543 nm (5% intensity), respectively. To separate the two fluorochromes, the emitted fluorescence was recorded at a BP of 488/543 nm and an LP of 585 nm in two different diachronic mirrors. For each biofilm sample, three (*x*: 230.34 μm, *y*: 230.34 μm, and *z*: 1 μm) dimensional stacks of horizontal plane images of areas were acquired. 3D projections of the biofilm structure were derived using the software Carl Zeiss Zen 3.3 (version Blue). The effects of postbiotics on the bacterial biofilm matrix were also visualised using scanning electron microscopy (SEM, Zeiss, EVO 40).^23^

### 2.7 Evaluation of anti‐QS activity

The expression of specific genes involved in the quorum‐sensing regulatory mechanisms of pathogens has been analysed by quantitative real‐time polymerase chain reaction (qRT‐PCR).^23^ Following the co‐incubation of pathogens with different concentrations of postbiotics, bacterial cells were harvested by centrifugation (9.000 × *g* for 10 min at 4°C), and total RNA was extracted using PureZOL™ RNA Isolation Reagent (Bio‐Rad, USA) according to the manufacturer's instructions. cDNA from RNA was then synthesised with an iScript cDNA Reverse Transcription Kit (Bio‐Rad, USA), and qRT‐PCR was conducted on a Bio‐Rad CFX96 Touch Real‐Time PCR system using an iTaq™ Universal SYBR® Green Supermix Kit (Bio‐Rad, USA) with the target gene primers (see Supplementary Table 1). The quantities of the target genes were expressed as the threshold number (*C*_*t*_) and were normalised relative to the 16S rRNA gene expression level as an internal control. The fold change in the expression of each gene was quantified according to the 2^−ΔΔ*Ct*^ method.

### 2.8 Determination of antioxidant activity

The phenolic content of the postbiotics was determined using the spectrophotometric method of Singleton and Rossi.^27^ Briefly, postbiotics were co‐incubated with Folin–Ciocalteu solution (50%, Sigma‐Aldrich, USA) at room temperature for 3 min in darkness. The colour change due to the reaction with sodium carbonate (2%, Merck, Germany) was then determined by measuring the absorbance at a wavelength of 750 nm. The results were evaluated in terms of gallic acid (GA, Sigma‐Aldrich, USA) equivalents (mg of GA/g) according to the calibration curve. To determine the flavonoid content, postbiotics were mixed with ethyl alcohol (96%, Merck, Germany), aluminium chloride (10%, Merck, Germany), and sodium acetate (1 M, Merck, Germany). After incubation in darkness at room temperature for 30 min, the absorbance of the solutions was measured at a wavelength of 415 nm. The flavonoid content of the postbiotics was calculated as quercetin equivalents (QE, Sigma‐Aldrich, USA) per gram weight of dry postbiotics according to the calibration curve.^28^

The ability of postbiotics to scavenge 2,2‐diphenyl‐1‐picrylhydrazyl (DPPH) free radicals was also assessed according to the method of Blois.^29^ Briefly, postbiotics at different concentrations were mixed with 120 μM DPPH (Sigma‐Aldrich, USA) in ethanol. Following the incubation in darkness at room temperature for 30 min, the absorbance was determined by spectrophotometric measurements at a wavelength of 415 nm. The percentage inhibition was calculated using the equation of [(*A*_control_ − *A*_sample_)/*A*_control_] × 100 (*A*: absorbance).

### 2.9 Cell viability assay

An MTT (3‐(4,5‐dimethylthiazol‐2‐yl)‐2,5‐diphenyltetrazolium bromide) assay was performed to determine the cytotoxicity of the postbiotics to cells. A total of 10,000 cells/well were seeded in 96‐well plates (Sarstedt, Germany) and allowed to attach for 24 h at 37°C with 5% CO_2_ and 95% relative humidity. Consequently, the cell culture medium was replaced with fresh DMEM containing different postbiotic concentrations. Untreated cells were used as a negative control. After 24 h of treatment, the cells were incubated with 10 μL of MTT (Sigma‐Aldrich, Germany) solution (5 mg/mL in PBS) for 4 h. The formazan crystals produced by the viable cells were then dissolved in 100 μL of dimethyl sulfoxide (DMSO, Serva, USA). The optical density of each well was measured at a wavelength of 570 nm using a microplate reader.

To quantify the live L929 cells treated with different concentrations of postbiotics, an Annexin V‐FITC Apoptosis Detection Kit (BioLegend, USA) was used according to the manufacturer's protocols. Briefly, L929 cells at a density of 5 × 10^5^ cells/well were cocultured with postbiotics in 6‐well plates (Sarstedt, Germany) for 24 h. After incubation, the cells were stained with FITC‐Annexin V and PI and then analysed by flow cytometry (NovoCyte ACEA, USA) using FlowJo software (version 10.5.3, Tree Star, San Carlos, USA).

### 2.10 Wound healing (Scratch) assay

To evaluate the wound‐healing capacity of the postbiotics, the scratch assay was performed according to the method of Liang et al.^30^ Cells were seeded in six‐well plates at a density of 1 × 10^6^ cells/well and incubated under standard conditions until they formed a confluent monolayer. Scratches were created by a sterile pipette tip across the centre of the well. Cellular debris was removed by washing the cells twice with PBS. The cells were then incubated with a fresh medium containing postbiotics. Untreated cells were used as a control. During the incubation period, the movement of the cells into the wound area was visualised under an inverted microscope (Olympus, Japan). The migration level between the two edges of the wound area was quantified by using ImageJ software (NIH, USA). The percentage of wound confluence was calculated using the equation of (*At*_0_ − *At*/*At*_0_) × 100, where *At*_0_ is the scratch area at time 0 h and *At* is the corresponding scratch area at different time intervals.

### 2.11 Analysis of collagen deposition

The expression of the collagen type I (*COL1A1*) gene was assessed by qRT‐PCR, essentially as previously described. Total RNA was extracted from L929 cells treated with or without postbiotics for 24 h, using TRIzol reagent. qRT‐PCR was performed on a Bio‐Rad CFX96 Touch Real‐Time PCR system using SYBR Green and the specific primers (see Supplementary Table 1). *GAPDH* was used as an internal control. In addition to gene expression analysis, collagen deposition was also assessed at the protein level by western blotting.^31^ Following the treatments, the cells were harvested and lysed with RIPA buffer for 30 min on ice. The supernatant was obtained by centrifugation (14,000 rpm for 30 min at 4°C), and the protein concentration was quantified with a BCA protein assay kit (Thermo Fisher Scientific, USA). Total proteins (25 μg/well) were separated by gel electrophoresis and transferred onto PVDF membranes using a Trans‐Blot SD semidry electrophoretic transfer system (Bio‐Rad, USA). The membranes were then incubated with primary antibodies against collagen type I (St John's Laboratory Ltd., UK) and *β*‐actin (Immunoway, YM3028) overnight at 4°C. HRP‐conjugated secondary antibody (goat antirabbit IgG conjugated, Santa Cruz, USA) was used and specific protein bands were visualised using an enhanced chemiluminescence system according to the manufacturer's instructions.

Immunofluorescence detection of COL1A1 was also investigated using fluorescence microscopy. Briefly, L929 cells grown on glass coverslips with or without postbiotics for 24 h were fixed in 4% paraformaldehyde (PFA, Sigma‐Aldrich, USA) in PBS for 20 min. The fixed cells were treated with a primary antibody against COL1A1 for collagen type I (green fluorescence) and then a secondary antibody with FITC as given above. DNA was stained with 4′,6‐diamidino‐2‐phenylindole (DAPI, Thermo Fisher Scientific, USA) for visualisation of cellular nuclei (blue fluorescence). Slides were mounted in ProLong Gold Antifade Reagent (Invitrogen, USA) and examined using a fluorescence microscope (Olympus BX60) equipped with a Color View III cooled CCD camera using an excitation wavelength of 488 nm and an emission wavelength of 520 nm.

### 2.12 Determination of intracellular reactive oxygen species (ROS) levels

Hydrogen peroxide (H_2_O_2_) was used for the induction of intracellular ROS levels in L929 cells. First, the effects of different H_2_O_2_concentrations on the L929 cell viability were determined by MTT assay, and the concentration that decreased the viability of L929 cells by 50% after 24 h of exposure was selected. Subsequently, intracellular ROS levels in H_2_O_2_‐challenged L929 cells treated with or without postbiotics were detected using a Cellular ROS Assay Kit (Abcam, UK) according to the manufacturer's instructions. Briefly, cells were cultured in six‐well plates at a density of 1 × 10^6^ cells/well and incubated under standard conditions. Following trypsinization and centrifugation as described above, the cells suspended in 100 μL of 25 μM 2,7‐dichloride‐hydro fluorescein diacetate (DCFH‐DA), a cell‐permeable probe, were incubated in darkness at 37°C for 30 min. Fluorescence, representing intracellular ROS levels, was measured at 485/20 nm excitation and 528/20 nm emission in a fluorescence multi‐detection reader using flow cytometry. The data were analysed using the FlowJo software.

### 2.13 Measurement of nitric oxide (NO) and immunological markers

Lipopolysaccharide (LPS) from *Escherichia coli* O111:B4 was used for the induction of NO and proinflammatory cytokine production in RAW 264.7 cells. First, the concentration of LPS which displayed non‐cytotoxicity on RAW 264.7 cells was determined by MTT assay. Subsequently, RAW 264.7 cells (2 × 10^5^ cells/well) were seeded into a 96‐well plate and treated with postbiotics with or without LPS for 24 h. After incubation, the culture supernatants were harvested, and the concentration of nitrite was determined using Griess reagent (2% sulfanilamide (w/v) in 5% phosphoric acid, 0.2% N‐(1‐naphthyl)ethylenediamine dihydrochloride in H_2_O (w/v)) [1:1]. The absorbance at 540 nm was measured using a microplate reader.^32^ The levels of cytokines IL‐6 and TNF‐*α* in the supernatant of RAW 264.7 cells subjected to various treatments were determined using commercially procured ELISA kits according to the manufacturer's protocol (ElabScience, USA). The quantities of cytokines secreted by RAW 264.7 cells were calculated using standard curves.

### 2.14 Characterisation of the metabolites in postbiotics

The phenolic and flavonoid profiles, along with the organic acids, fatty acids, and vitamins present in the postbiotics, were quantified using chromatographic techniques as described in the methodology outlined by Sevin et al.^33^ All analyses were performed at the Erciyes University Technology Research and Application Center (Turkey). Organic acids, phenolic and flavonoid components were identified and quantified using high‐performance liquid chromatography (HPLC; Shimadzu Prominence Series‐LC‐20A, Kyoto, Japan), with standard calibration curves derived from certified reference standards. Fatty acids analysis based on fatty acid methyl esters (FAMEs) was conducted using gas chromatography–mass spectrometry (GC–MS) methods. Additionally, a comprehensive multi‐vitamin analysis, encompassing both water‐soluble and fat‐soluble vitamins, was performed using a highly sensitive and selective high‐performance liquid chromatography–tandem mass spectrometry (LC–MS/MS) platform.

### 2.15 Statistical analysis

All assays were performed with three independent experiments, and each measurement was conducted in triplicate. Data were analysed using SPSS version 22.0 (IBM, New York, NY, USA) and GraphPad Prism v.3.0 (GraphPad Prism v.3.0, GraphPad Software, San Diego, CA, USA). The student's *t*‐test was used to compare the means between two groups, while analysis of variance (ANOVA) was used to compare the means among three or more groups. All results were presented as the mean ± standard deviation, and *p* < 0.05 was considered to indicate a significant difference.

## 3 RESULTS

### 3.1 Selection of the candidate strain

**FIGURE 1:**
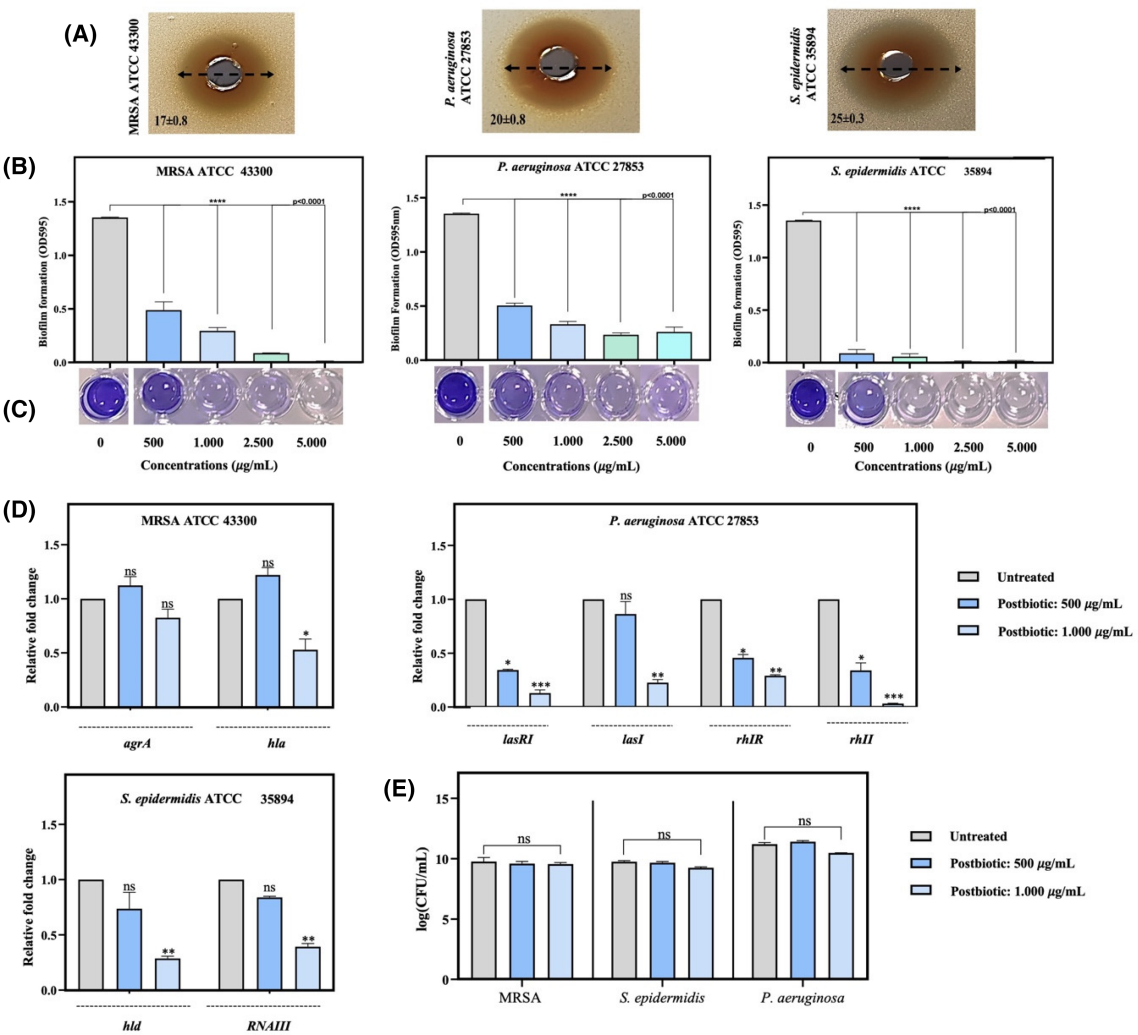
Inhibitory effects of postbiotics against skin pathogens (A) Inhibition zones (mm) one agar plates (B, C) Biofilm formation displayed with crystal violet assay (D) Relative gene expression of quorum sensing‐related genes and (E) the cell viability of pathogens in the different groups of treatments. Data are presented as the mean ± SD (*n* = 3). Statistical significance was determined using the following symbols: *, *p* < 0.05; **, *p* < 0.01; ***, *p* < 0.001; ****, *p* < 0.0001, n.s., non‐significant.

The candidate strain for postbiotic production was selected based on its antimicrobial activity against important skin pathogens. Following the isolation process, three colonies per animal displaying phenotypic characteristics similar to those of *Lactobacillus* spp. on species‐specific MRS agar media were chosen. Eighteen putative isolates with no catalase activity but with Gram‐positive reactions were screened by evaluating the antagonistic activity of their postbiotics against *P. aeruginosa* PAO1/ATCC 27853, MRSA ATCC 43300, and *S. epidermidis* ATCC 35894 using an agar well diffusion assay. Our results indicated that the postbiotics from 8 of the 18 isolates showed no inhibition, while 10 inhibited at least one or two pathogenic strains (data not shown). Among them, one postbiotic derived from a rod‐shaped and Gram‐positive isolate exhibited a broader and stronger antagonistic activity against all tested pathogens, with radii of inhibition zones ranging from 17 to 25 mm, and was selected for further analysis. The highest antibacterial activity was observed with an inhibition zone of 25 ± 0.3 mm against *S. epidermidis* ATCC 35894 (Figure 1A). No inhibition was observed in the MRS medium, which was used as a negative control. The antagonistic effects of the postbiotics against skin pathogens were also analysed to determine the components responsible for the antibacterial activity, such as organic acids, hydrogen peroxide, or bacteriocin‐like compounds. Although the inhibitory effect was not mediated by proteinase K or catalase treatment, the activity disappeared after neutralisation, likely due to acidity, as the postbiotics had a pH of 4.11. Additionally, the MICs of the postbiotics against skin pathogens were determined to be 25 mg/mL for *S. epidermidis* ATCC 35894 and 40 mg/mL for the other two tested pathogens.

The selected isolate, with its strong antimicrobial activity, was then identified using *16S rRNA* gene sequencing (1.492 bp) followed by a BLAST search against the GenBank Bacteria and Archaea 16S ribosomal RNA sequence databases. According to the results obtained, the isolate matched most closely with *L. reuteri* with 100% similarity. It was registered in the National Centre for Biotechnology Information (NCBI) as *L. reuteri* EIR/Spx‐2 (EIR: the code of our culture collection, Spx: abbreviation of *Spalax*, 2: the second isolate of the collection) under the accession number PP951925.

### 3.2 Anti‐biofilm and anti‐QS activity against skin pathogens

The anti‐biofilm efficacy of postbiotics against skin pathogens was assessed using a crystal violet assay. Our findings indicated that postbiotic concentrations ranging from 500 to 5.000 μg/mL were significantly effective in inhibiting biofilm formation by all tested pathogens (Figure 1B, C). Consistent with the antibacterial activity results, the highest anti‐biofilm activity was observed against *S. epidermidis* ATCC 35894. Specifically, a concentration of 1.000 μg/mL reduced the biofilm of *S. epidermidis* ATCC 35894 by 91.44% ± 3.11%, whereas reductions of 87.21% ± 2.88% and 79.44% ± 1.94% were noted for *P. aeruginosa* PAO1/ATCC 27853 and MRSA ATCC 43300, respectively. Based on the significant results (*p* < 0.0001), the two lowest concentrations (500 and 1.000 μg/mL) were selected for further analysis.

Subsequently, the impact of postbiotics on gene expression related to the major QS signal transduction system and virulence was evaluated by qRT‐PCR assay (Figure 1D). In MRSA ATCC 43300, co‐treatment with the selected postbiotic concentrations did not result in a significant change in *agrA* gene expression. However, the concentration of 1.000 μg/mL significantly downregulated the expression of the *hla* gene (1.89‐fold, *p* < 0.05). For *P. aeruginosa* PAO1/ATCC 27853, a substantial decrease in the expression of *lasRI* (7.7‐fold, *p* < 0.001), *lasI* (4.35‐fold, *p* < 0.01), *rhIR* (3.45‐fold, *p* < 0.01), and *rhII* (33.3‐fold, *p* < 0.001) genes was observed following 24 h of treatment with 1.000 μg/mL postbiotic. Additionally, *hld* (3.45‐fold, *p* < 0.01) and *RNAIII* (2.5‐fold, *p* < 0.01) were significantly downregulated in *S. epidermidis* ATCC 35894 in a dose‐dependent manner compared to those in the untreated group. Importantly, the downregulation of these target genes did not compromise cell viability, as determined by CFU counting (Figure 1D), indirectly demonstrating that the postbiotics exhibited anti‐QS activity even at non‐lethal concentrations.

**FIGURE 2:**
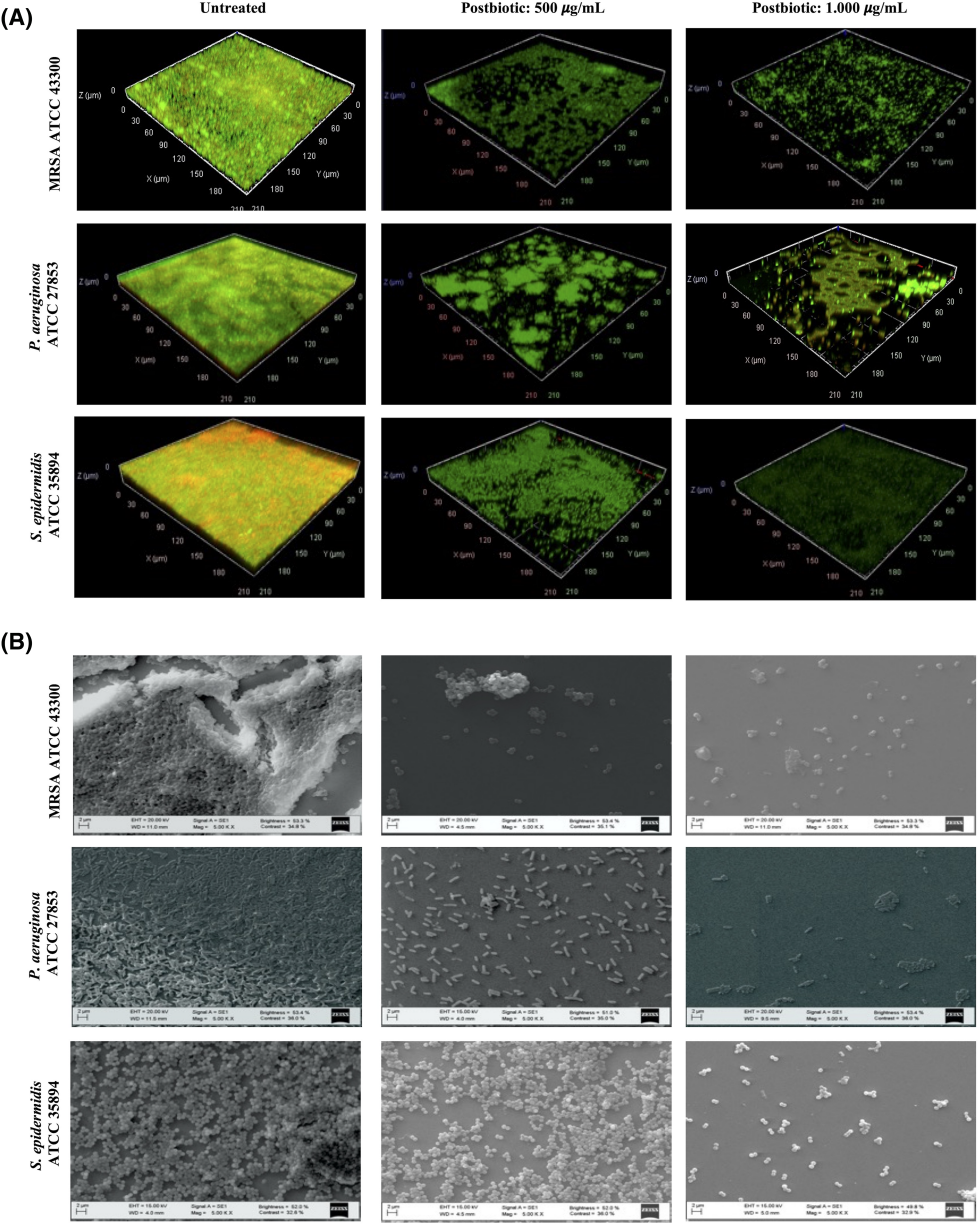
(A) Confocal laser microscopy images and (B) scanning electron micrographs illustrating the effect of postbiotics on the cell viability of pathogens and their biofilm formations in the different groups of treatments.

Microscopic examinations further elucidated the structural organisation of biofilms formed by skin pathogens and confirmed the inhibitory effects of postbiotics on biofilm formation. CLSM analysis revealed that biofilm production was highly intense in untreated groups after 24 h. However, following postbiotic treatment, there was an increase in the number of dead cells labelled with PI (red) and a decrease in the number of live cells stained with SYTO 9 (green) (Figure 2A). Additionally, SEM analysis of biofilm architecture demonstrated substantial biofilm production and the presence of adhering bacterial cells in untreated samples. Biofilms in the untreated groups appeared as multilayered aggregates of cells with extracellular matrix deposition surrounding the cell clusters. In contrast, postbiotic treatment significantly reduced biofilm intensity with diminished bacterial cell attachment to the surface in a dose‐dependent manner (Figure 2B).

### 3.3 Antioxidant activity

The antioxidant activity of the postbiotics was assessed based on their ability to scavenge DPPH radicals. The results revealed that a concentration of 500 μg/mL of postbiotics achieved a DPPH scavenging activity of 72% ± 4.02%, while a concentration of 1.000 μg/mL resulted in complete (100%) inhibition. Furthermore, the antioxidant potential of postbiotics was evaluated in terms of the phenolic and flavonoid contents using spectrophotometric methods. The findings indicated that postbiotics derived from the EIR/Spx‐2 strain exhibited a high phenolic content of 7.12 mg GAE/g and a substantial flavonoid content of 4.8 mg QE/g.

### 3.4 L929 cell proliferation

**FIGURE 3:**
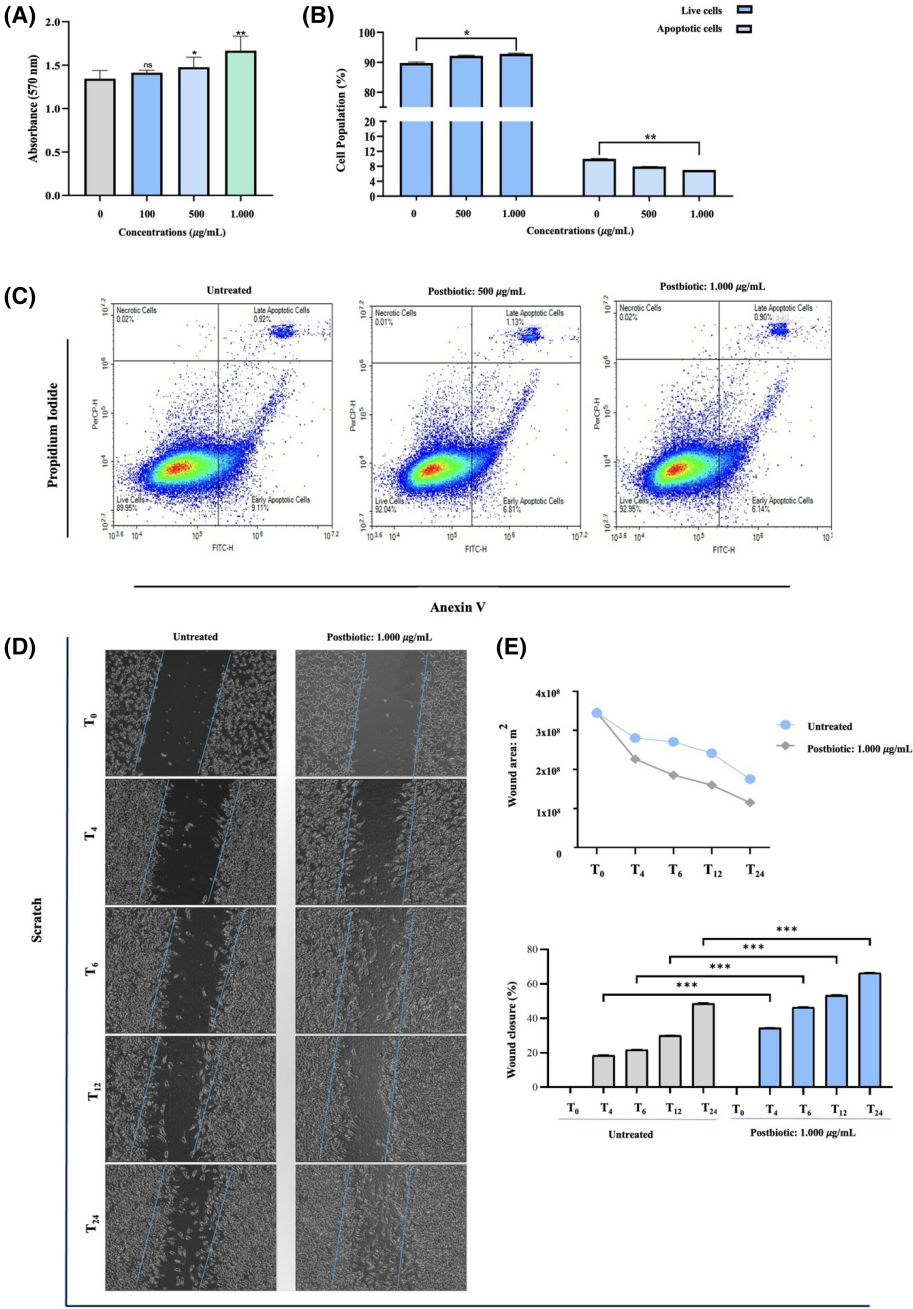
(A) Cell viability of L929 cells upon treatment with postbiotics determined by (A) MTT assay and (B) flow cytometry analysis using Annexin V‐FITC/PI staining (C) The quadrants define necrotic (single PI‐positive) cells, late apoptotic cells (annexin V and PI double‐positive), early apoptotic cells (annexin V single‐positive), and healthy cells (nonapoptotic cells) (D) Photomicrographs of L929 cells during in vitro wound‐healing/scratch assay at the indicated time points using a 10× objective on an inverted microscope (E) Wound area and wound closure (%) of each treatment group at 24 h of postinjury. Data are presented as the mean ± SD (*n* = 3). Statistical significance was determined using the following symbols: *p < 0.05, **p < 0.01, ***p < 0.001, n.s., non‐significant.

To evaluate the effects of postbiotics on the viability of L929 fibroblastic cells, an MTT assay was conducted. The results indicated that a postbiotic concentration of 500 μg/mL (*p* < 0.05) and 1.000 μg/mL (*p* < 0.01) significantly increased L929 cell viability by 10.04% ± 1.04% and 23.82% ± 2.11%, respectively, compared to the untreated group (Figure 3A). Since the MTT assay only measures cell viability or cytotoxicity at a single time point, additional experiments utilising Annexin V‐FITC/PI staining were performed to gain a more comprehensive understanding of the proliferative effects of postbiotics on L929 cells. The results revealed a significant increase in the proportion of live L929 cells (*p* < 0.05) when treated with 1.000 μg/mL of postbiotics (Figure 3B), as indicated by the lower left quadrant of the flow cytometry plots, which represent viable cells negative for Annexin‐FITC and PI (Figure 3C). Additionally, a significant reduction (*p* < 0.01) in the number of apoptotic cells was observed in cultures treated with 1.000 μg/mL of postbiotics (Figure 3B). It is noteworthy that postbiotic treatment did not induce significant necrosis compared to untreated cells. Based on these findings, the 1.000 μg/mL postbiotic concentration, demonstrating more pronounced effects than the 500 μg/mL, was selected for further assays.

### 3.5 Accelerated migration of L929 cells

The effect of postbiotics on the migration ability of L929 cells was evaluated using a scratch assay. After 24 h of co‐treatment, microscopic observation revealed that the scratch wound in the treated wells was significantly filled with cells due to accelerated cell proliferation, compared to the untreated samples (Figure 3D). The initial scratch area at *T*_0_ (negative control) was measured as 3.32 × 10^8^ ± 0.14 μm^2^ using the wound healing size tool plugin of ImageJ software. At 24 h post‐injury, the wound area had reduced to 1.25 × 10^8^ ± 0.09 μm^2^, indicating a significant relative wound closure of 66.78% ± 3.74% (*p* < 0.05) compared to the controls. In contrast, untreated cells achieved only 48.94% ± 2.51% closure of wound area (Figure 3E).

### 3.6 Collagen deposition in L929 cells

**FIGURE 4:**
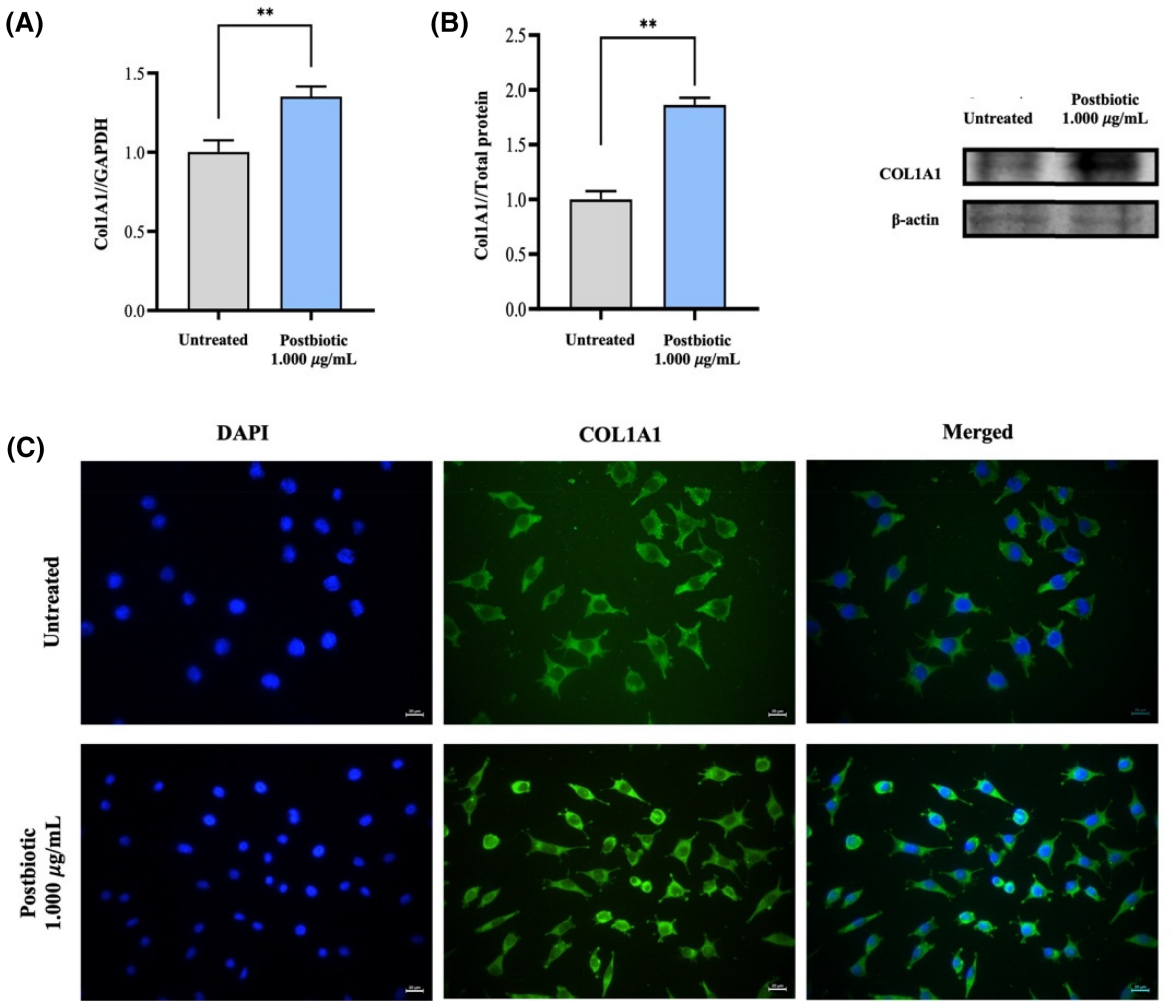
Collagen type 1 (COL1A1) production in fibroblast cell line L929 when treated with postbiotics. (A) Gene expressions determined by qRT‐PCR analysis. (B) Protein fold‐change evaluated by western blotting. (C) Immunofluorescence detection investigated by fluorescence microscopy (COL1A1, green fluorescence; DAPI for cellular nuclei, blue fluorescence.

The effects of postbiotics on type I collagen synthesis in fibroblasts were assessed through qRT‐PCR and western blot analyses. Treatment of L929 cells with 1.000 μg/mL of postbiotics for 24 h resulted in a significant upregulation of COL1A1 mRNA expression by 1.35‐fold (*p* < 0.01) compared to untreated cells (Figure 4A). Western blot analysis further confirmed a significant increase in the COL1A1 protein level by 1.86‐fold (*p* < 0.01) in postbiotic‐treated L929 cells relative to untreated cells (Figure 4B). Additionally, immunofluorescence analysis of COL1A1 production in both untreated and postbiotic‐treated L929 cells is depicted in Figure 4C. The micrographs indicated an increased number of L929 cells in the postbiotic‐treated group, which may contribute to the elevated levels of COL1A1.

### 3.7 Intracellular ROS accumulation in L929 cells

**FIGURE 5:**
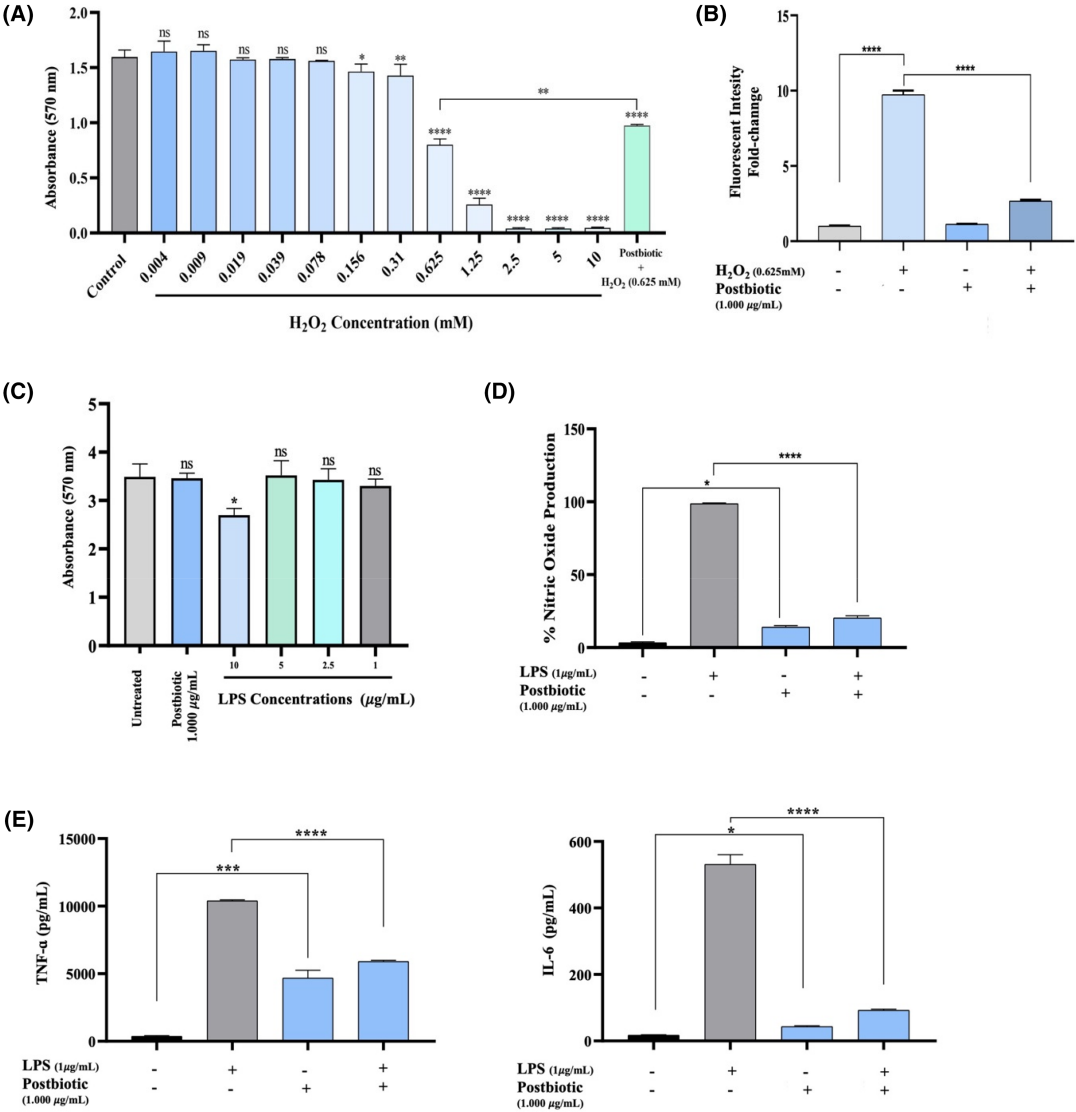
(A) Cell viability of L929 cells upon treatment with different H_2_O_2_ concentrations with or without postbiotics determined by MTT assay. (B) Fold chance of fluorescent intensity of intracellular ROS determined by flow cytometry. (C) Cell viability of RAW264.7 murine macrophages cells upon treatment with different LPS concentrations and postbiotics determined by MTT assay (D) NO (E) TNF‐*α* and IL‐6 productions in LPS‐induced RAW264.7 murine macrophages with or without probiotics. Data are presented as the mean ± SD (*n* = 3), Statistical significance was determined using the following symbols: *, *p* < 0.05; **, *p* < 0.01; ***, *p* < 0.001; ****, *p* < 0.0001, n.s., non‐significant.

To induce oxidative stress in L929 cells, a concentration of 0.625 mM H_2_O_2_ was selected, which reduced the viability of L929 cells by 50.04% ± 2.01% after 24 h of exposure. Notably, postbiotic treatment significantly (*p* < 0.01) improved the viability of H_2_O_2_‐induced L929 cells compared to untreated H_2_O_2_‐induced L929 cells (Figure 5A). Subsequently, the effects of postbiotics on H_2_O_2_‐induced L929 cells were evaluated by flow cytometry analysis using DCFDA dye. Based on our results, 0.625 mM H_2_O_2_ concentration resulted in a significant increase in intracellular ROS levels (9.73‐fold, *p* < 0.0001). However, postbiotic co‐treatment for 24 h resulted in a 3.66‐fold decrease in intracellular ROS levels compared to untreated H_2_O_2_‐induced L929 cells (*p* < 0.0001). Notably, postbiotic treatment alone did not significantly alter the intracellular ROS levels in L929 cells (Figure 5B).

### 3.8 Effects on the viability of RAW264.7 murine macrophages

Preliminary cytotoxicity tests were conducted to determine the non‐cytotoxic concentration of LPS for treating macrophages. The effects of LPS at various concentrations on RAW264.7 cell viability were assessed using an MTT assay. The results indicated that the lowest concentration of LPS (1 μg/mL) did not significantly affect macrophage viability and was thus selected for further experiments (Figure 5C). Moreover, a cell viability assay was also performed to rule out any potential cytotoxicity of the selected postbiotic concentration on RAW264.7 cells. Consistent with previous findings, a postbiotic concentration of 1.000 μg/mL did not adversely affect the viability of RAW 264.7 cells (Figure 5C).

### 3.9 NO production in LPS‐induced RAW264.7 murine macrophages

Following 24 h of co‐treatment with LPS (1 μg/mL) and postbiotics (1.000 μg/mL), NO levels in the RAW 264.7 cell culture media were measured using spectrophotometric analysis. The results showed that LPS (1 μg/mL) significantly (*p* < 0.0001) increased NO production in RAW 264.7 cells. In contrast, postbiotic treatment markedly reduced NO levels by 79.25% ± 3.18% in LPS‐stimulated RAW 264.7 cells compared to untreated LPS‐stimulated cells, which exhibited 100% NO production. Furthermore, postbiotic treatment did not induce NO production in macrophages (Figure 5D).

### 3.10 Anti‐inflammatory effects on RAW264.7 murine macrophages

The anti‐inflammatory potential of postbiotics from the EIR/Spx‐2 strain was further evaluated in vitro using LPS‐induced RAW 264.7 murine macrophages. The effect of postbiotics on the transcriptional levels of the TNF‐*α* and IL‐6 cytokine genes, which are involved in the inflammatory response, was assessed by qRT‐PCR analysis. Our results indicated that LPS, as a pro‐inflammatory stimulus, significantly induced the transcription of TNF‐*α* (28.5‐fold induction, *p* < 0.0001) and IL‐6 (30.83‐fold induction, *p* < 0.0001) in untreated RAW264.7 cells. In contrast, treatment with postbiotics markedly attenuated the levels of these pro‐inflammatory cytokines in LPS‐stimulated RAW264.7 cells. Specifically, postbiotics significantly reduced TNF‐*α* levels by 1.76‐fold compared to the elevated secretion of TNF‐*α* in untreated LPS‐stimulated RAW 264.7 cells (*p* < 0.0001). IL‐6 transcription was also reduced by 5.77‐fold in postbiotic‐treated macrophages relative to LPS‐stimulated macrophages without postbiotic treatment. Notably, although postbiotics increased IL‐6 and TNF‐α production in unstimulated RAW264.7 cells (*p* < 0.05 and *p* < 0.001), the magnitude of this effect was found significantly lower compared to that induced by LPS (*p* < 0.001) (Figure 5E).

### 3.11 Metabolites in postbiotics

Chromatographic analyses were performed to determine various organic acids, FAMEs, vitamins, phenolic compounds, and flavonoids in the postbiotics. Among the organic acids, lactic acid was the most abundant (24.62 mg/mL), followed by butyric acid (5.45 mg/mL). The FAMEs profile revealed significant concentrations of methyl stearate (C18:0; 91 mg/mL), methyl palmitate (C16:0; 46 mg/mL), and methyl oleate (C18:1 cis‐9; 8 mg/mL). Vitamin analysis indicated a high abundance of B‐complex vitamins, particularly B12 (398.72 μg/L) and B5 (78.14 μg/L), along with moderate levels of vitamins E1 (51.71 μg/L) and B1 (54.36 μg/L). Phenolic and flavonoid profiling highlighted gallic acid (25.4 mg/L), catechin (12.6 mg/L), and epicatechin (9.6 mg/L) as the most prominent compounds, along with syringic acid, p‐coumaric acid, and kaempferol (see Supplementary Table 2).

## 4 DISCUSSION

The term ‘wound’ is defined as the loss of integrity and normal function of the skin due to injury or surgical operations.^34^ Depending on the management, wounds are classified into acute wounds, which typically heal within 7–10 days, and chronic wounds, which fail to progress normally due to various environmental and host‐related factors such as infection, prolonged inflammation, and oxidative stress associated with ageing.^34^ Therefore, the comprehensive treatment of chronic wounds is a significant unmet medical need due to the complexity of the wound microenvironment.^7^ In any case, to pave the way for the promotion of wound healing, an exhaustive understanding of the healing process and the use of potential bioactive compounds that benefit each healing phase are considered important. Unfortunately, there is still no universally effective treatment method that addresses all phases of wound healing. Consequently, with the increasing demand for modern treatment strategies, the development of alternative bioactive compounds has recently become a popular topic in the wound care industry.

Postbiotics, the newest member of the biotic family, are valuable functional bioactive substances produced by probiotics. They have recently attracted a great deal of interest due to their numerous beneficial characteristics, which contribute to the acceleration of each phase of wound healing.^34^ Based on the current state of evidence, postbiotics which exhibit immunomodulatory, anti‐inflammatory, antimicrobial, antioxidant, and angiogenic effects effectively boost the wound healing process.^35^ To date, several probiotics and their postbiotics derived from either human origin or dairy‐associated environments have been characterised for their wound‐healing properties.^35^ In the present study, *L. reuteri* strain EIR/Spx‐2 was used as a novel postbiotic source. This strain was isolated for the first time from the gut microbiota of long‐lived blind mole rats, as the gut microbiota has recently attracted increasing attention as a modulator of healthy ageing.^36^ Recent studies have demonstrated that the molecular and cellular hallmarks of ageing in mammals identified at a fundamental level^37^ are accompanied by changes in the microbiota, which, in turn, affect the rate of age‐related decline. Therefore, microbiota‐based ageing interventions, such as probiotics and postbiotics, have gained importance for preventing ageing‐linked physiological decline facilitated by dysbiotic microbiota.^36^ It is noteworthy that ageing is also associated with impairment of wound healing, which significantly hinders the skin's healing function by prolonging the inflammatory phase and increasing oxidative stress.^38^ From this perspective, we propose that the gut microbiota of blind mole rats may contribute to their extraordinary longevity and healthy ageing. Thus, focusing on their microbiota should be a key goal of microbiota‐associated therapeutic interventions aimed at wound healing. In this regard, this study was undertaken to evaluate the effects of postbiotics from the EIR/Spx‐2 strain on various dynamic biological and physiological processes of wound healing under in vitro conditions.

The initial phase of wound repair, haemostasis, is highly susceptible to infection due to the ability of microbial cells to easily penetrate the damaged skin barriers and invade the surrounding tissues.^12^ The microorganisms most commonly present at the onset of wound infection are Gram‐positive bacteria, such as *S. aureus* and *Enterococcus* spp., while Gram‐negative organisms, such as *E. coli* and *P. aeruginosa*, typically appear in later stages.^6^ Antibiotics have been widely used for centuries to treat wound infections caused by these pathogens. However, antibiotic resistance, which is prevalent among these pathogens, and the detrimental effects of antibiotics on the pre‐existing normal skin microbiota, remain significant issues, prompting the search for alternative strategies.^7^, ^12^ Over the past few decades, non‐antibiotic components such as silver and plant‐based products have garnered increased attention for combating wound infections.^7^ Additionally, recent alternatives include bacteriophage therapy, antimicrobial peptides, oral and topical probiotics or their derivatives, antibody therapy, and antibacterial nanomaterials, all of which offer a more bioactive approach to facilitate wound healing.^39^ One of the essential properties of postbiotics is their antimicrobial activity, making them potential therapeutic candidates against wound infections.^34^ In our study, postbiotics derived from the EIR/Spx‐2 strain exhibited a broad range of inhibitory activity against the most common skin pathogens often implicated in chronic infections. Furthermore, the antimicrobial activity was inhibited by neutralisation, suggesting that the lowered pH of postbiotics, related to the production of organic acids, is responsible for this inhibition. While acidity can negatively affect many pathogens by disrupting their cellular processes or inhibiting their growth, commensal microbes may be more resilient due to their evolutionary adaptations to acidic conditions. This balance allows the body to control harmful pathogens while maintaining beneficial microbes, which play important roles in health and immune regulation.^40^ Similar to our findings, postbiotics derived from *L. plantarum* F‐10, isolated from the faecal microbiota of healthy breastfed infants, inhibited the growth of *P. aeruginosa* PAO1/ATCC 27853, MRSA ATCC 43300, and their hospital‐derived strains isolated from chronic skin wound samples.^26^ However, no inhibition was observed when the postbiotics were neutralised. This mode of action against pathogens can be attributed to acetic and lactic acid in the postbiotics produced by lactic acid bacteria, which affect the enzyme activity of pathogens and cause the bacterial cell to expend all their energy to release extra protons (H^+^), ultimately resulting in cell death.^41^ In contrast to our results, strains such as *L*. *rhamnosus* GG and *L*. *casei* Shirota display antimicrobial activity not attributed to acid,^42^ bacteriocin, or hydrogen peroxide production,^43^ highlighting the diversity of protective mechanisms of postbiotics.

Pathogens commonly associated with chronic wound infections can form biofilms that are highly resistant to conventional antibiotherapies and host immune responses.^6^ Thus, the formation of bacterial biofilms is critically considered a further paradigm shift in clinical wound care strategies. Consequently, we investigated the antibiofilm effects of postbiotics derived from the EIR/Spx‐2 strain. Our findings indicated that postbiotics significantly inhibited biofilm formation by skin pathogens. In addition, CSLM and SEM micrografts revealed the alterations in biofilm structure and clustering behaviour of the pathogens. Recent studies have shown that postbiotics with their bioactive substances exhibit their antibiofilm effects by disrupting the bacterial cell communication of pathogens known as the QS mechanism.^44^, ^45^ From this point of view, the anti‐QS activity of postbiotics from the EIR/Spx‐2 strain was also evaluated by qRT‐PCR analysis. The results demonstrated significant downregulation of the genes *LasI/R and RhlI/R*, which are key components of the synthase/transcription factor receptors known as two N‐acyl homoserine lactone (AHL) systems that regulate biofilm formation and virulence in *P. aeruginosa*.^46^ *LasR*, a master transcriptional regulator of QS that upregulates *rhlI*/*R*, was also downregulated, highlighting the importance of postbiotics in infection progression. Additionally, the transcriptional level of the hemolysin gene *hla* (*α*‐hemolysin), crucial for MRSA virulence,^47^ was also reduced upon treatment with postbiotics. While limited experimental evidence exists regarding the anti‐QS effect of postbiotics, our findings align with previous studies showing that postbiotics from *L. crustorum* ZHG 2‐1, a novel QS bacterium, reduced the expression of the QS‐regulated genes *lasI/R* and *rhlI/R*.^48^ Furthermore, *L*. *plantarum* supernatant disrupts the pathogenic properties of *P. aeruginosa* by interfering with its QS system.^49^ Collectively, our results suggest that postbiotics inhibit the growth of skin pathogens through pH‐dependent mechanisms and reduce biofilm formation and cellular communication by decreasing the expression of virulence genes. This could represent a novel and effective strategy for controlling skin pathogens in infected wounds.

Oxidative stress induced by high levels of ROS is a major factor contributing to chronic wounds. To maintain non‐toxic levels of ROS in wound areas, antioxidants that can donate electrons to ROS or transform ROS into stable molecules are therapeutic targets for regulating the healing process.^50^ In particular, the antioxidant mechanisms of postbiotics include scavenging oxidant compounds, reducing activity, chelating metal ions, and preventing intestinal ROS formation.^51^ The present study revealed that the use of postbiotics potentially enhances antioxidant activity by increasing the scavenging of free radicals. One explanation for the remarkable antioxidant effects of our native postbiotic may be attributed to its high content of flavonoids and phenolic compounds. Recent studies have also demonstrated that acetic acid, the predominant component of the postbiotic derived from the strains of *Lactobacillus* spp. and *Bifidobacterium* spp., may be responsible for significant antioxidant activity.^52^ In addition to antioxidant activity determined by spectrophotometric measurements, the intracellular activity of our postbiotics was also confirmed using L929 fibroblast cells. Our results also showed that postbiotics protected skin cells from H_2_O_2_‐induced oxidative stress by reducing intracellular ROS levels. Similarly, postbiotics derived from probiotic strains (*Lc. lactis* MG5125, *B. bifidum* MG731, and *B. lactis* MG741) alleviated H_2_O_2_‐induced oxidative stress in HepG2 cells by mediating lipid peroxidation and glutathione.^49^ Moreover, *B. bifidum* exerts antioxidant activity by reducing oxidant radicals,^53^ and *B. lactis* has been reported to exhibit antioxidant effects by producing metabolites such as folic acid and butyrate.^54^

The inflammatory phase is another crucial process in the wound‐healing cascade.^7^ During this phase, immune cells, including neutrophils and macrophages, release soluble mediators such as proinflammatory cytokines (IL‐1*β*, IL‐6, IL‐8, and TNF‐*α*) and growth factors that orchestrate tissue repair by removing harmful microorganisms and cell debris from damaged tissues, eventually activating the fibroblasts needed in the subsequent phase.^12^ Importantly, prolonged inflammation can lead to pathological conditions that impair the wound‐healing process.^7^ Therefore, compounds with anti‐inflammatory activity are considered important therapeutic targets. In this present study, the immunomodulatory effects of postbiotics on macrophages were investigated. Our results showed that postbiotic treatment modulated the cytokine patterns in macrophages challenged with LPS in vitro by attenuating the secretion of TNF‐*α* and IL‐6 in response to a pro‐inflammatory signal. Consistent with these findings, recent studies with probiotics and postbiotics have also demonstrated their anti‐inflammatory effects. For instance, *L. plantarum* as whole cells downregulated the transcription of TNF‐*α* and IL‐8 in LPS‐stimulated macrophages.^55^, ^56^ Additionally, postbiotics from various lactic acid bacteria were found to significantly induce IL‐10 secretion by PBMC‐derived macrophages before or after the LPS challenge.^55^, ^57^ Indeed, the immunomodulating effect of postbiotics on LPS‐stimulated macrophages could be attributed to competition for the activation of the same inflammatory pathways. For instance, postbiotics from *L. plantarum* have been shown to induce cytokine production in THP‐1 macrophages by activating Toll‐like receptor 2 (TLR‐2) in a species‐ and strain‐dependent manner.^58^

During the proliferation and remodelling phases of wound repair, fibroblasts proliferate and migrate into the wound bed, and produce extracellular matrix components such as collagen to restore the epithelial barrier.^12^ Hence, compounds that promote these phases in the healing process play a crucial role.^7^ In our study, postbiotics exerted wound‐healing effects by increasing fibroblast cell proliferation and migration, and promoting type I collagen synthesis, overall favouring the remodelling phase of the wound‐healing process. Consistent with our findings, *L*. *rhamnosus* GG and *L*. *reuteri* have been shown to enhance re‐epithelialization by increasing both keratinocyte migration and proliferation.^59^, ^60^ However, the two postbiotics were not equally effective at promoting cell migration, with *L. rhamnosus* GG being more effective than *L. reuteri*, highlighting the strain‐specific effects of postbiotics. Researchers have investigated the mechanisms that could improve cell migration and proliferation, suggesting increased expression of growth factors and extracellular matrix components.^61^ In this regard, aside from enhancing cell proliferation and migration, our postbiotics also influenced collagen deposition. As an important structural protein in the skin, type I collagen is synthesised primarily by dermal fibroblasts.^62^ Therefore, the proliferation of fibroblasts promoted by postbiotics might also support type I procollagen synthesis. Additionally, the attenuation of the pro‐inflammatory state by postbiotics might also be related to collagen deposition in fibroblasts.^62^ Moreover, several studies have evaluated type I procollagen production by fibroblast cells upon treatment with various strains of lactic acid bacteria, such as *B. animalis* ssp. *lactis* MG741^63^ and *Lactobacillus* spp., *Streptococcus* spp., and *Bifidobacterium* strains^64^ in search of anti‐ageing benefits of bacteria via the gut‐skin axis.^62^

In conclusion, the outcomes of this study indicate that postbiotics from the EIR/Spx‐2 strain can effectively participate in all four stages of the wound healing process by reducing oxidative stress through the generation of intracellular ROS, modulating the immune response by decreasing pro‐inflammatory cytokines, enhancing cell proliferation and migration for tissue regeneration, and reducing the likelihood of infection by eliminating invading pathogens and their biofilms. The diverse metabolite profile of postbiotics displayed a strong positive correlation with their biological activity, highlighting their potential as a valuable resource for wound healing applications. Among these metabolites, lactic acid has the ability to inhibit pathogenic bacteria while fostering a favourable environment for beneficial microbes.^39^ It also supports skin health by enhancing hydration and maintaining pH balance, making postbiotics particularly relevant for dermatological and wound‐healing purposes.^65^ Similarly, formic acid exhibits antibacterial properties, particularly against biofilm‐forming bacteria,^66^ which could improve the efficacy of postbiotics in antimicrobial formulations. Gallic acid and p‐coumaric acid, both potent antioxidants and antimicrobial agents,^67^ may protect cells from oxidative stress and combat infections at wound sites. Flavonoids such as catechin, epicatechin, and kaempferol exhibit strong antioxidative and anti‐inflammatory properties,^68^ promoting tissue regeneration. Regarding fatty acids, methyl stearate may aid in restoring the skin barrier and improving hydration, while methyl oleate, as an unsaturated fatty acid, demonstrates anti‐inflammatory properties,^69^ further supporting the regenerative and dermatological applications of postbiotics. Among the detected vitamins, B12 plays a critical role in cellular repair and immune system support,^70^ while B5, essential for coenzyme A synthesis, enhances wound healing by promoting skin regeneration and reducing inflammation.^71^ Additionally, vitamin E1, a potent antioxidant, protects cellular membranes from oxidative stress, supporting skin healing and exhibiting anti‐ageing effects.^72^ These findings underscore the rich biochemical composition of postbiotics, highlighting their multifunctional bioactivity. Further research is needed to fully explore their metabolites for therapeutic and industrial applications, particularly in wound care and beyond.

## AUTHOR CONTRIBUTIONS

*Conceptualization*: F.K. (lead). *Data curation*: F.K. (lead), H.K.D. (equal), Z.B.A. (equal), B.K. (equal). *Formal analysis*: F.K. (lead). *Funding acquisition*: F.K. (lead). *Investigation*: F.K. (equal), H.K.D. (equal), Z.B.A. (equal), B.K. (equal), T.K. (equal), K.C.A. (equal). *Methodology*: F.K. (lead), H.K.D. (equal), Z.B.A. (equal), B.K. (equal), T.K. (equal), K.C.A. (equal). *Supervision*: F.K. (lead), K.C.A. (supporting). *Validation*: F.K. (equal), T.K. (equal), K.C.A. (equal). *Visualisation*: F.K. (lead), H.K.D. (equal), Z.B.A. (equal), B.K. (equal). *Writing‐original draft preparation*: F.K. (lead), H.K.D. (equal), Z.B.A. (equal), B.K. (equal). *Writing‐review and editing*: F.K. (lead), H.K.D. (equal), Z.B.A. (equal), B.K. (equal), T.K. (equal), K.C.A. (equal).

## CONFLICT OF INTEREST STATEMENT

Authors declare no conflicts of interest.

## Supporting information


**Data S1**.

## Data Availability

The data that supports the findings of this study are available from the corresponding author upon reasonable request.
