# Supplementary material for: In vitro wound healing effects of postbiotics derived from the gut microbiota of long‐lived blind mole rats, a model of healthy ageing
Source: Wound Repair Regen. 2025 Apr 18;33(2):e70023. doi: 10.1111/wrr.70023 (PMC12006833; doi:10.1111/wrr.70023)
Supplement: Supplementary file 1 — Data S1. [file WRR-33-0-s001.docx]

**SUPPLEMENTARY FILE**

**Table 1.** Sequences of primers used in this study

| **Genes** | **Primer Sequences (5’-3’)** | |
| --- | --- | --- |
|  | **Forward** | **Reverse** |
| ***agrA*** | CTACAAAGTTGCAGCGATGGA | TGGGCAATGAGTCTGTGAGA |
| **hla** | ACAATTTTAGAGAGCCCAACTGAT | TCCCCAATTTTGATTCACCAT |
| **hld** | TGAGTTGTTGAGCCATCCCA | ACGTAACACTGAGTCCAAGGAA |
| **RNAIII** | ACTAAATCACCGATTGTAGAAATGATATCT | ATTTGCTTAATCTAGTCGAGTGAATGTTA |
| ***lasI*** | CGCACATCTGGGAACTCA | CGGCACGGATCATCATCT |
| ***lasR*** | CTGTGGATGCTCAAGGACTAC | AACTGGTCTTGCCGATGG |
| ***rhlI*** | GTAGCGGGTTTGCGGATG | CGGCATCAGGTCTTCATCG |
| ***rhlR*** | GCCAGCGTCTTGTTCGG | CGGTCTGCCTGAGCCATC |
| ***rpoD***  (*P. aeruginosa)* | TCCATCGCAAGAAGTAACAC | TTGTAGCCACGACGGTATTC |
| **16S rRNA**  (MRSA) | TAACTTCGGGAAACCGGAGC | GCATCGTTGCCTTGGTAAGC |
| **16S rRNA**  (*S. epidermidis)* | TACACACCGCCCGTCACA | CTTCGACGGCTAGCTCCAAAT |
| **16S rRNA**  (universal) | AGAGTTTGATCCTGGCTCAG | GGTTACCTTGTTACGACTT |
| ***COL1A1*** | GACGCCATCAAGGTCTACTG | ACGGGAATCCATCGGTCA |
| **GAPDH** | TGCCCAGAACATCATCCCTG | GACGGACACATTGGGGGTAG |

**Table 2.** Metabolites detected in postbiotics

| Organic acids (mg/mL) |  |
| --- | --- |
| Maleic acid | 3.21 |
| Tartaric acid | 1.39 |
| Lactic acid | 24.62 |
| Formic acid | 1.46 |
| Acedic acid | 0.87 |
| Butyric acid | 5.45 |
| FAMEs (mg/mL) |  |
| C16:0 methyl palmitate | 46 |
| C18:0 methyl stearate | 91 |
| C18:1 cis-9 methyl oleate | 8 |
| Vitamins (μg/L) |  |
| B1 | 54.36 |
| B2 | 35.95 |
| B3 | 2.58 |
| B5 | 78.14 |
| B12 | 398.72 |
| D2 | 0.19 |
| D3 | 0.68 |
| E1 | 51.71 |
| K3 | 7.58 |
| Phenolic and flavonoids (mg/L) |  |
| Syringic Acid | 3,9 |
| p-Qumaric Acid | 0,8 |
| Gallic acid | 25,4 |
| Catechin | 12,6 |
| GalloCatechin | 7,8 |
| EpiCatechin | 9,6 |
| Kaempferol | 3,3 |

**Figure 1.** Karyotype of *Nannospalax xanthodon* (2n=58)

**
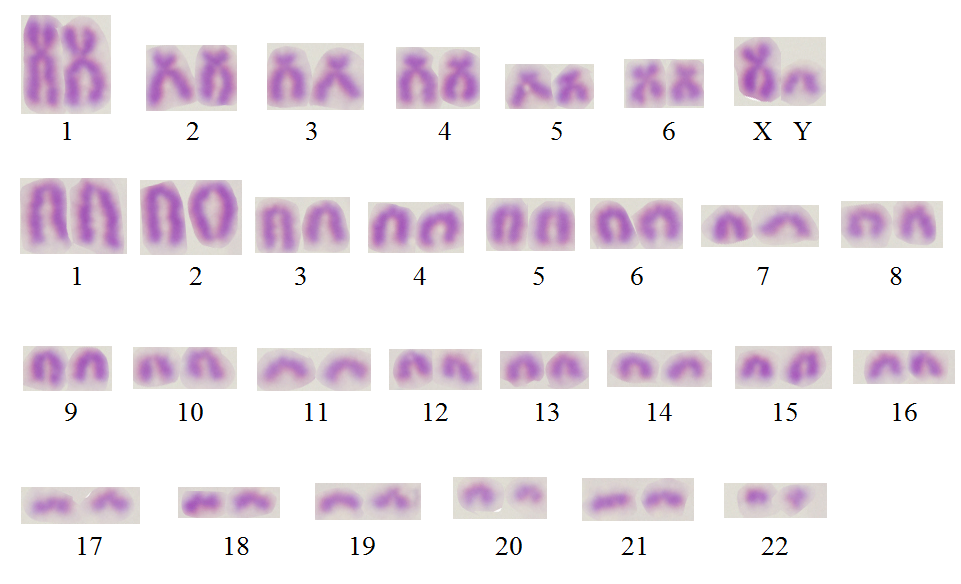
**
